# Supplementary material for: Structure and mechanism of the RalGAP tumor suppressor complex
Source: Nat Commun. 2025 Jul 30;16:7002. doi: 10.1038/s41467-025-61743-9 (PMC12311180; doi:10.1038/s41467-025-61743-9)
Supplement: Supplementary file 1 — Supplementary Information [file 41467_2025_61743_MOESM1_ESM.pdf]

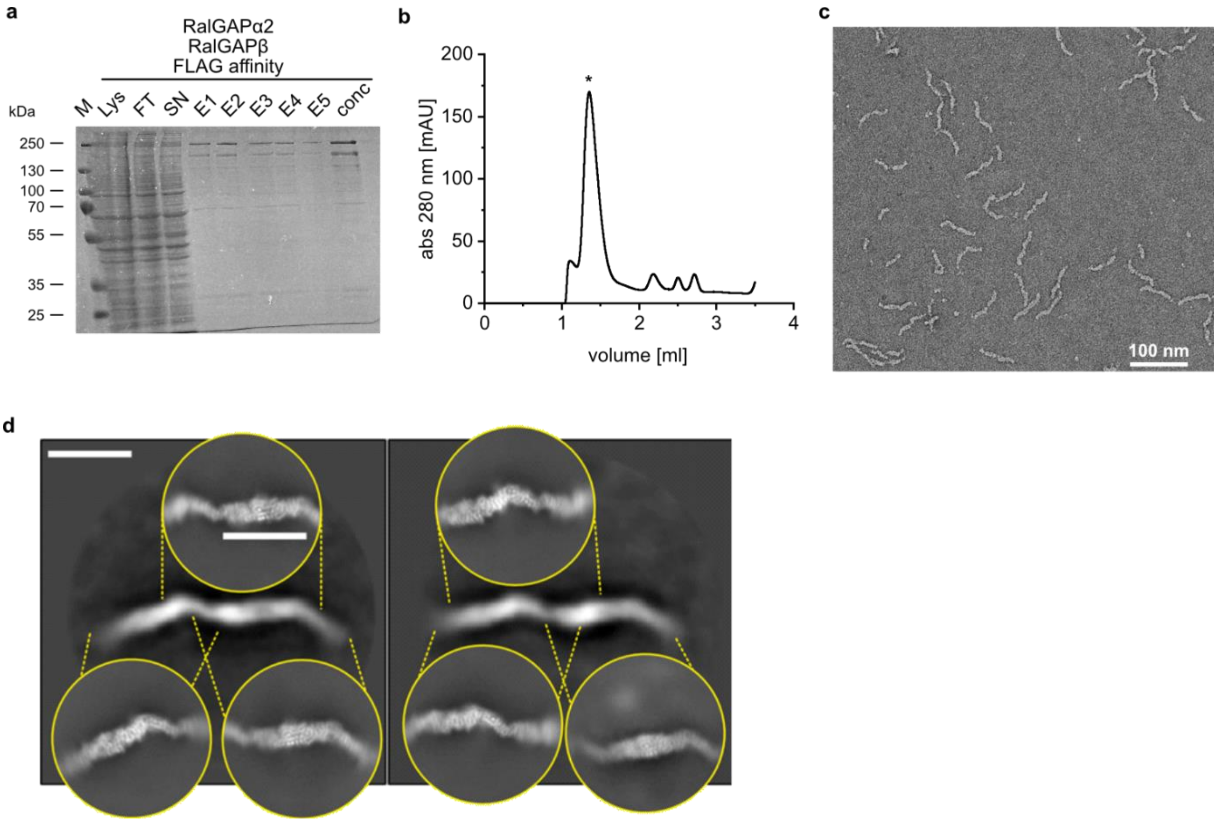

Supplementary Figure 1: Purification of RalGAP. a Coomassie stained SDS-PAGE gel of a RalGAP FLAG affinity purification. **b** Size exclusion chromatography profile of RalGAP (Superose 6 increase 5/150 column (Cytiva), 20 mM HEPES pH 7.5, 150 mM NaCl, 2 mM $MgCl_2$, 1 mM TCEP). Peak fraction used for EM analysis is marked by an asterisk. **c** Representative negative stain EM micrograph of RalGAP preparation. Scale bar is 100 nm. **d** Representative reference-free 2D class averages with a box size of 1440 pixels (binned to 360 pixels with pixel size of 2.32 Å) is shown and compared with class averages from particles that were used for the final reconstructions, with box size of 720 pixels (binned to 360 pixels with pixel size of 1.16 Å). Scale bars are 20 nm.

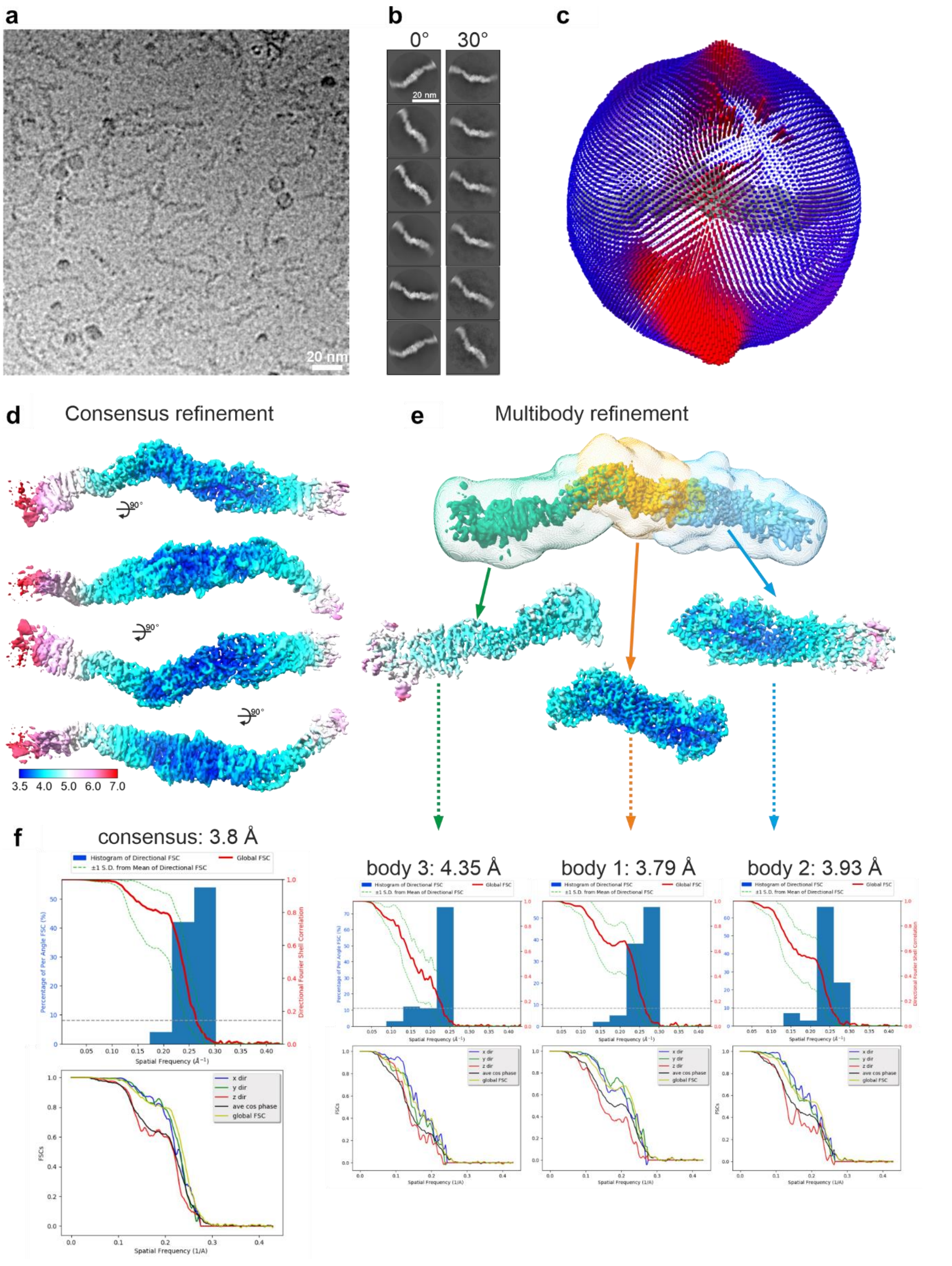

Supplementary Figure 2: Cryo-EM analysis of RalGAP. a Representative cryo-EM micrograph. **b** Representative reference-free 2D class averages from datasets collected at 0° and 30°, respectively. **c** Angular distribution of RalGAP particles in the final consensus refinement. **d** Density maps of the RalGAP consensus refinement colored by local resolution prior multi-body refinement. **e** The used masks for Relion5 multibody-refinement and the generated locally refined maps colored by local resolution. **f** 3D-FSCs of the consensus and masked maps.

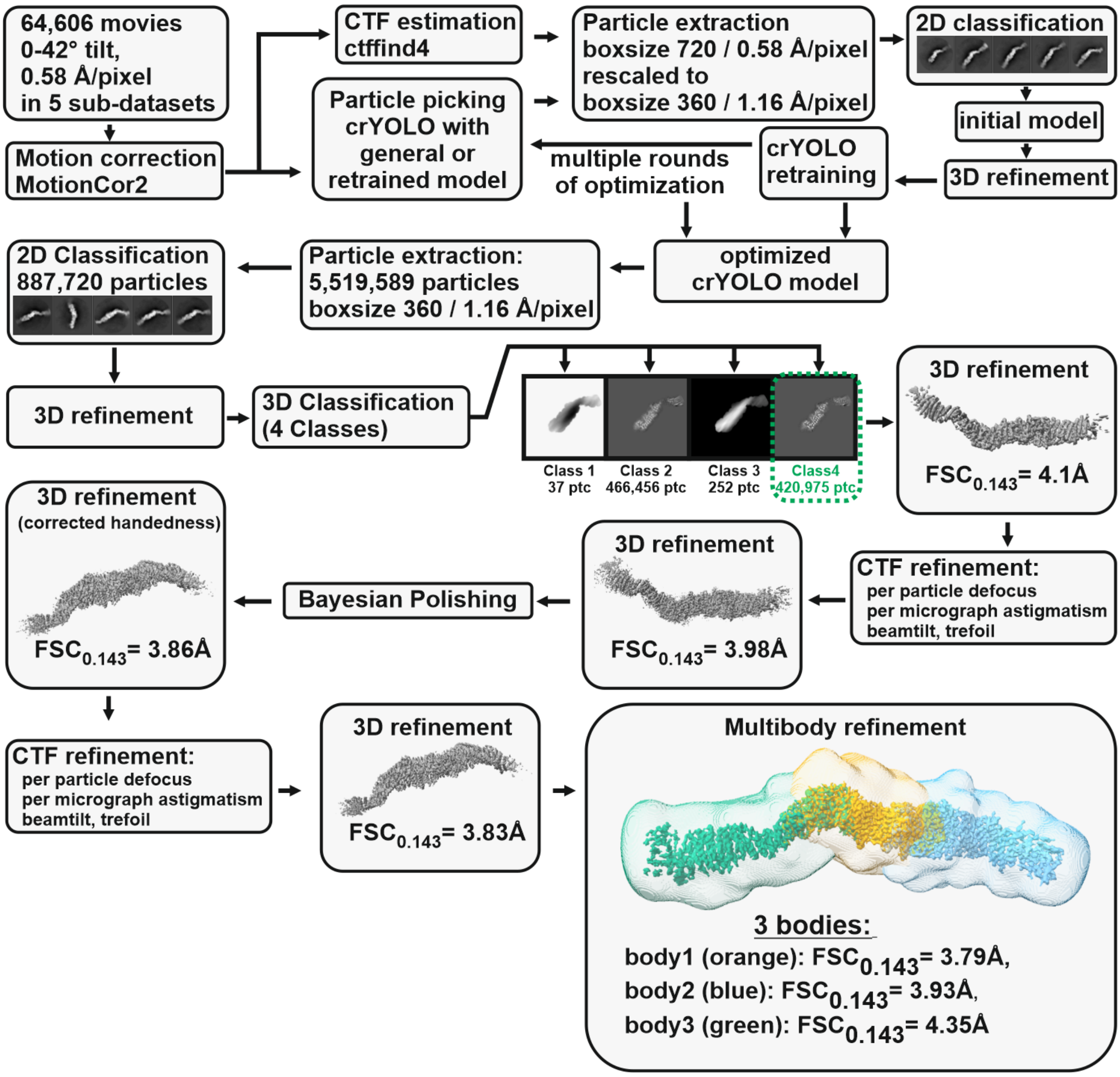

Supplementary Figure 3: Cryo-EM processing workflow for structure determination of RalGAP.

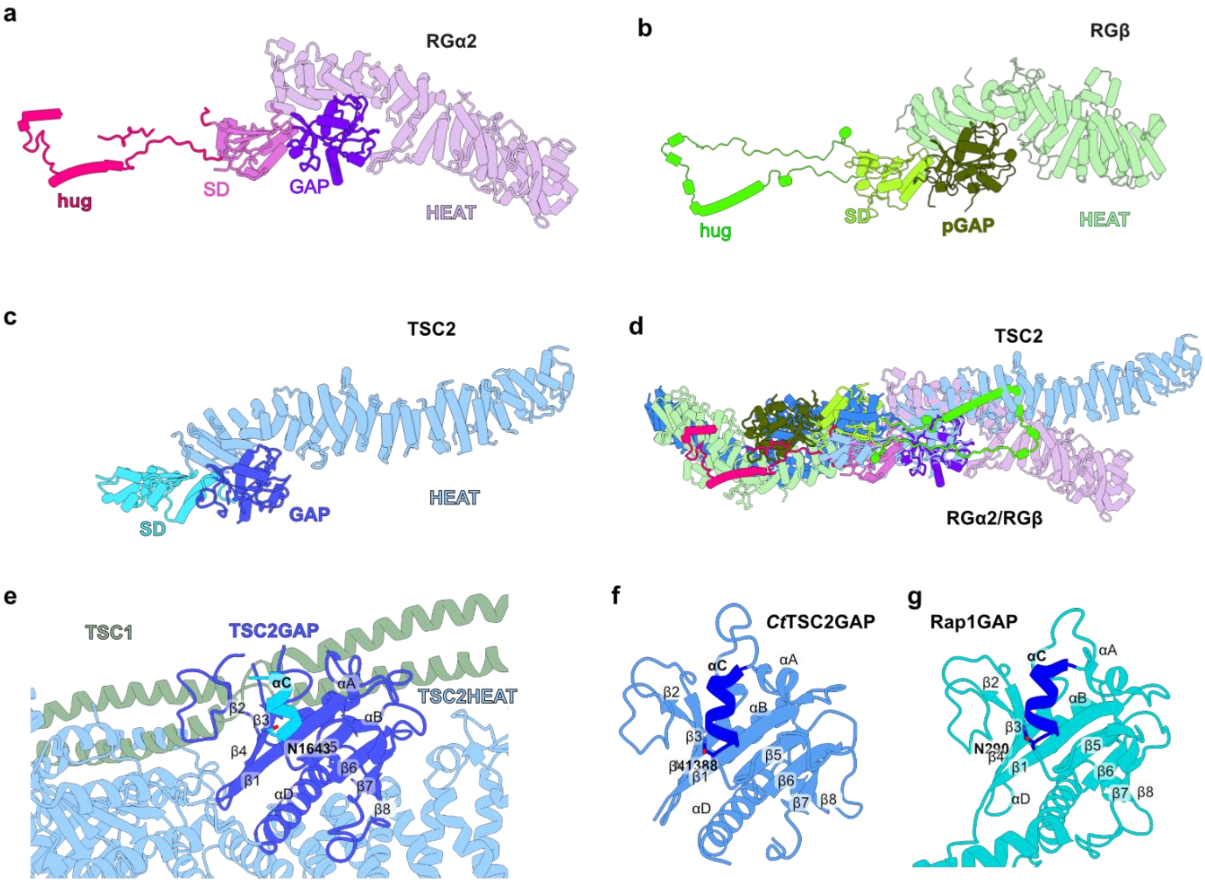

Supplementary Figure 4: Structural comparison of RalGAP and the TSC complex. a Structures of RGα2, **b** RGβ, and **c** TSC2 with the SD and (p)GAP domains in the same orientation **d** Superposition of the RalGAP heterodimer and TSC2 homodimer. **e** Experimental structures of Asn-Thumb GAP domain of human TSC2 in the TSC complex (PDBID: 7DL2)[1], **f** the isolated TSC2 GAP domain of *Chaetomium thermophilum* (PDBID: 6SSH)[2], and **g** Rap1GAP (PDBID: 3BRW)[3] with the catalytic helices αC and the catalytic asparagine highlighted.

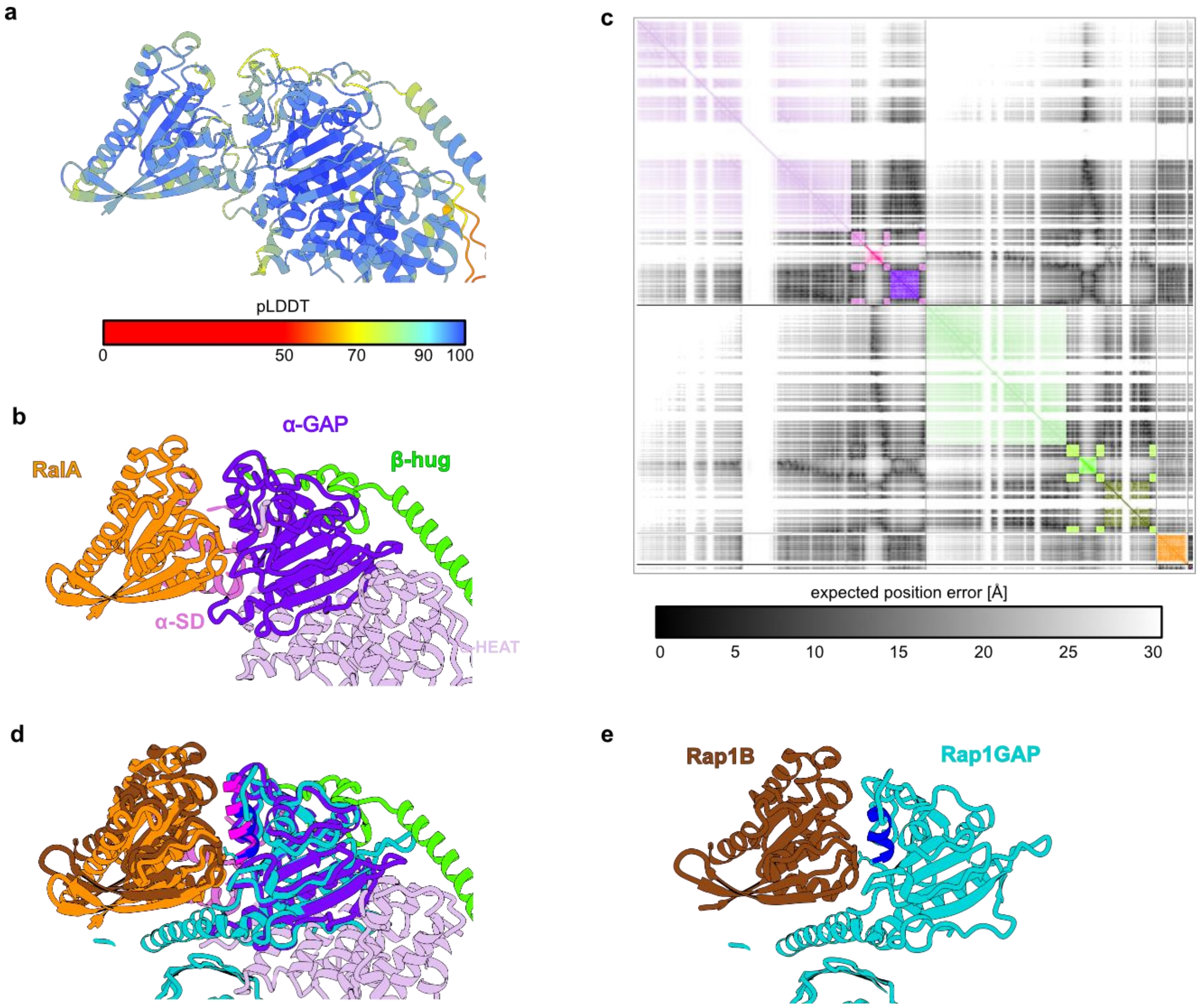

Supplementary Figure 5: Modeling of the interaction between Ral and RalGAP. a AlphaFold3 model of RalA interaction with RGα2/RGβ colored by pLDDT (predicted local distance difference test) score and **b** colored by domains. **c** Error plot of the RalA/RGa2/RGβ AlphaFold3 model with domain colors. **d** Superposition of the RalA/RalGAP complex model with the Rap1B/Rap1GAP structure. **e** Crystal structure of the Rap1B/Rap1GAP complex (PDBID: 3BRW).

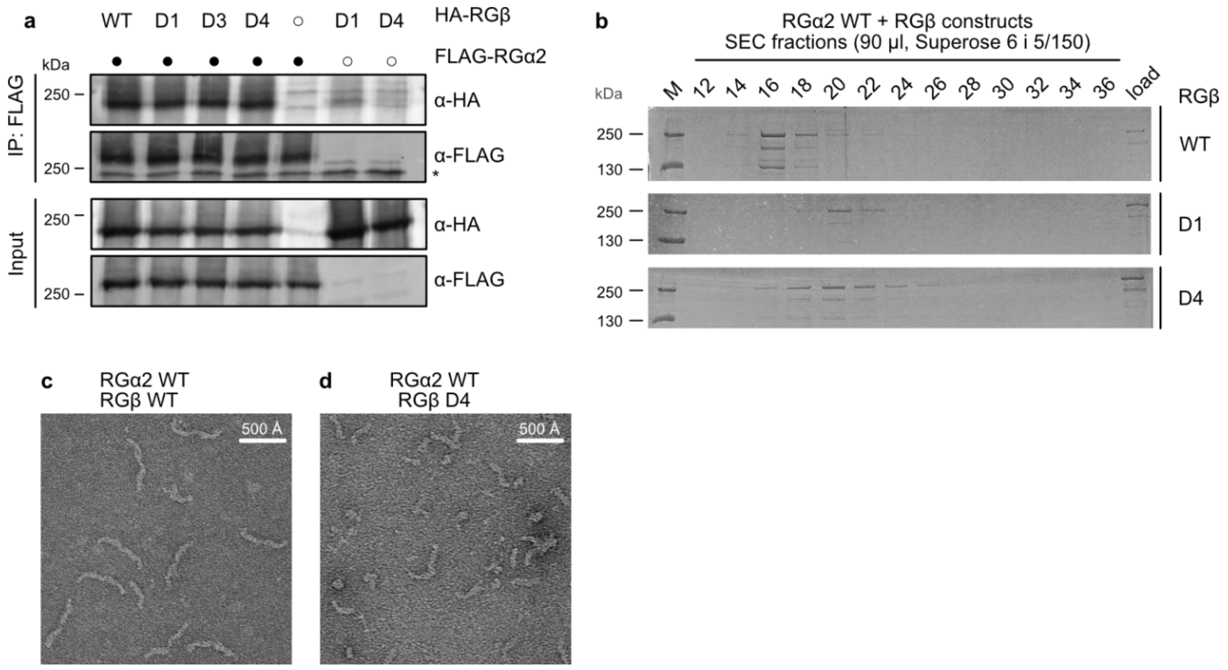

Supplementary Figure 6: Homodimerization of RGβ. a Co-immunoprecipitations of RGα2 and RGβ variants from transiently transfected HEK293FT cells. D1: W65R; D3: V29E, V33Q, V37E; D4: V29E, V33Q, V37, W65R; WT: wild-type. Results from representative example of three independent repeats. **b** Analysis of RalGAP complexes with RGβ WT and RGβ mutants with size exclusion chromatography (Superose 6 increase 5/150 column (Cytiva), 20 mM HEPES pH 7.5, 150 mM NaCl, 2 mM $MgCl_2$, 1 mM TCEP). **c, d** Representative negative stain images of purified RGα2/RGβ WT and D4 mutant.

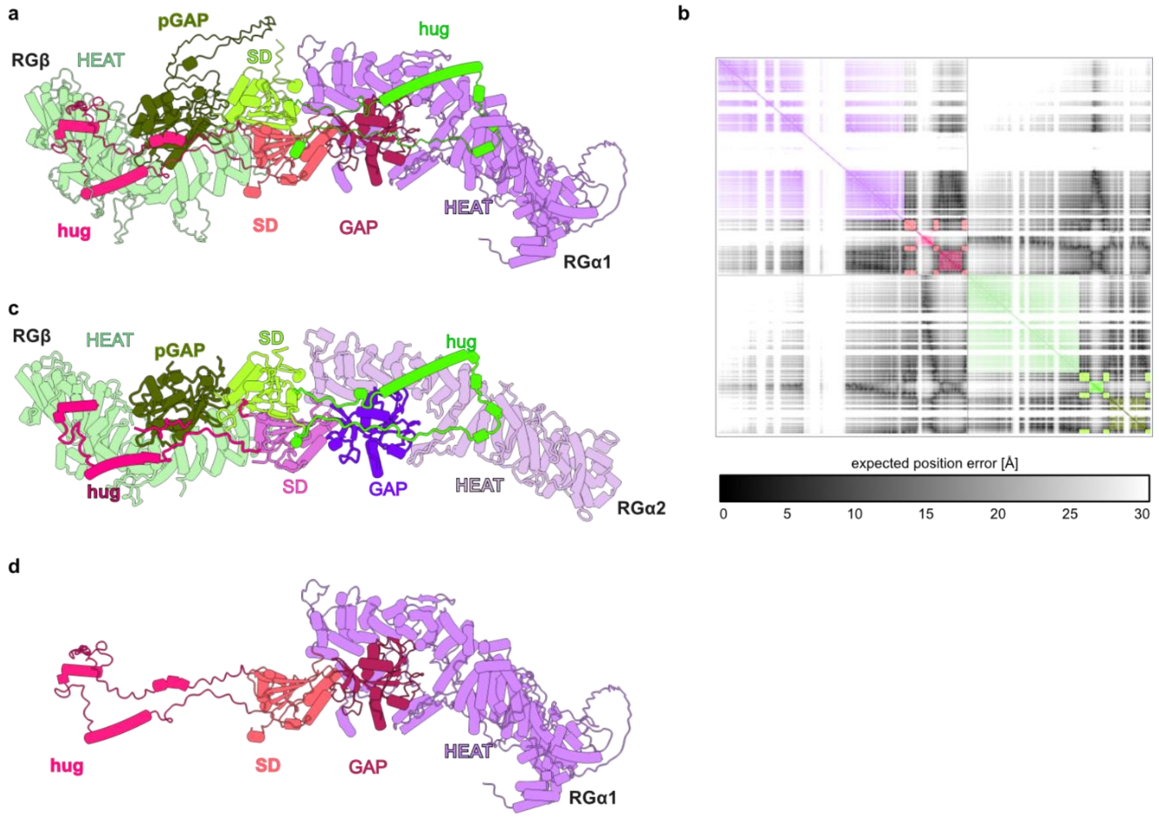

Supplementary Figure 7: Modeling of the RGα1/RGβ complex. a AlphaFold3 model of the RGα1/RGβ complex colored by domains. Extended disordered loop regions are removed from the representation. **b** Error plot of the RGa1/RGβ AlphaFold3 model with domain colors. **c** Experimental structure of RGα2/RGβ. **d** Predicted structure of RGα1 in the RalGAP complex.

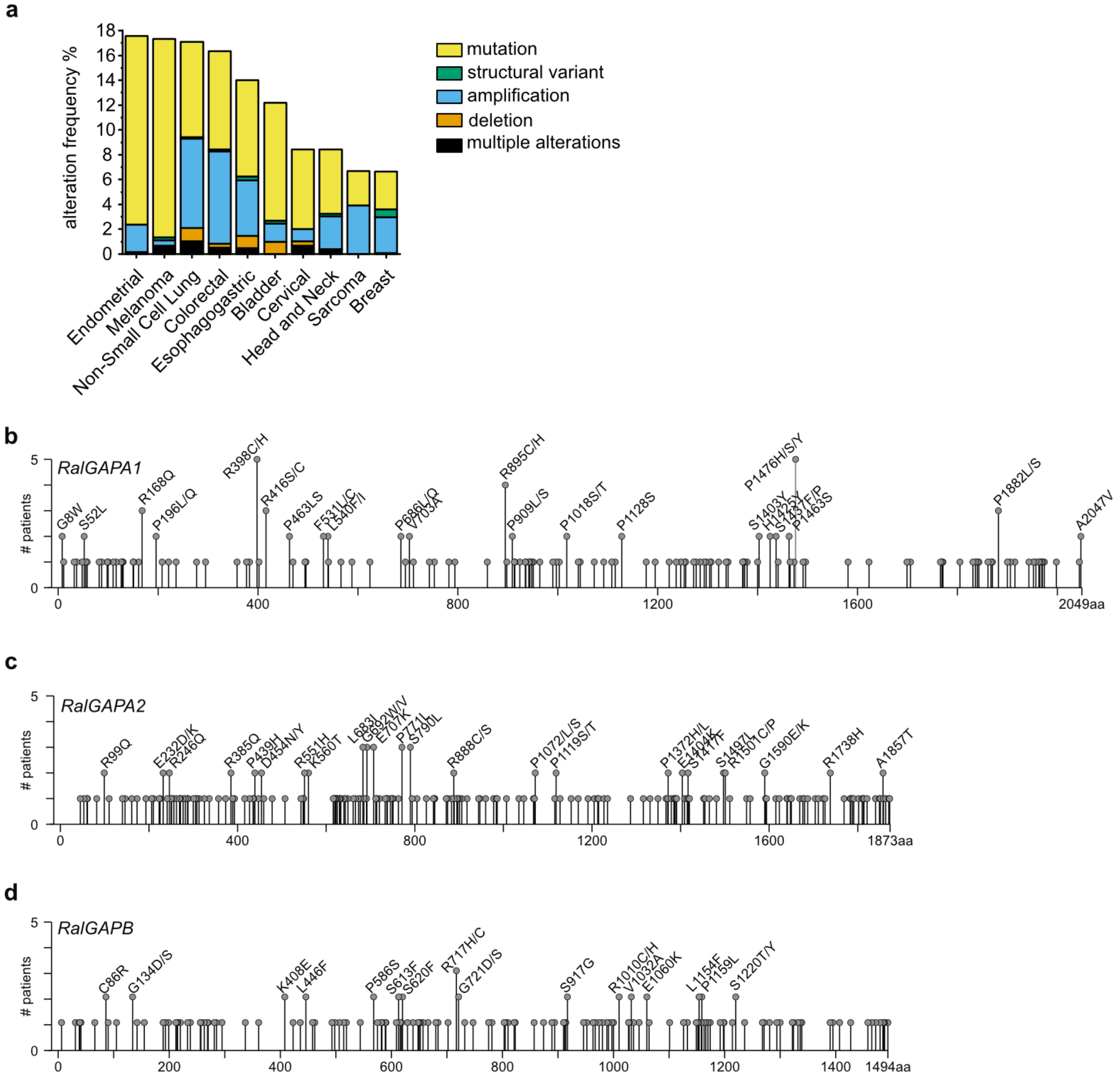

Supplementary Figure 8: Distribution of *RALGAP* variants reported in cancer patients. **a** Summary of alterations in the *RALGAPA1*, *RALGAPA2* and *RALGAPB* genes reported in the TCGA PanCancer Atlas Studies. The ten cancer types with the highest alteration frequency are shown. **b** Lollipop plots of variants of the *RALGAPA1*, **c** *RALGAPA2*, and **d** *RALGAPB* genes reported in samples form patients with uterine and skin cancer. Generated with cBioportal[4,5] and modified.

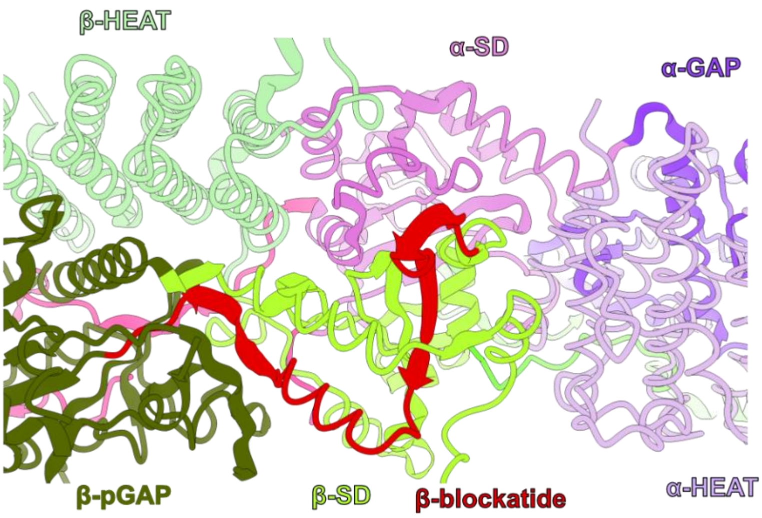

Supplementary Figure 9: Mapping of the β-blockatide peptide in the RalGAP structure.

Supplementary table 1: Cryo-EM data collection and refinement statistics of RalGAP.

**Data collection**			
Microscope	Titan Krios G4 (Selectris X, E-CFEG)		
Voltage (kV)	300		
Camera	Falcon 4i		
Pixel size (Å)	0.58		
Tilt angle	all Micrographs	picked Micrographs	Particles initial/final
0°	7,239	5,935	719,364 / 90,076
0°	4,136	3,978	381,698 / 63,172
30°	3,770	3,231	304,681 / 40,685
0°	40,001	27,246	3,814,505 / 214,791
42°	9,445	2,733	299,341 / 12,251
total number of particles	5,519,589		
Number of frames	793-819		
Number of fractions	61-63		
Total electron dose (e^-/Å^2)	60		
Defocus range (µm)	-0.5 – -4.2		
**Atomic model composition**			
Chains	4		
Symmetry imposed	C1		
Non-hydrogen (protein) atoms	26999		
Residues	3394		
particle substack	420,975		
Ligand atoms	-		
**Refinement (Phenix)**	Composite map		
RMSD bond (Å) (# > 4σ)	0.002 (0)		
RMSD angle (°) (# > 4σ)	0.481 (5)		
Model to map fit, CC mask	0.79		
Model to map fit, CC box	0.77		
Resolution (FSC@0.143, Å)	3.4 (masked)<br>3.7 (unmasked		
B-factor (mean, Å^2)	129.72		
**Validation**			
Clashscore	7.67		
Ramachandran outliers (%)	0.00		
Ramachandran allowed (%)	3.42		
Ramachandran favoured (%)	96.58		
Molprobity score	1.64		
EMRinger score	1.241		

Supplementary table 2: RGα2 and RGβ variants from cBioportal. Missense variants reported more than once in uterine and skin cancer patients are listed with the AlphaMissense classification and a structural assessment based on the experimental cryo-EM structure of the RalGAP complex.

Variant	AlphaMissense pathogenicity score		Structural assessment
RalGAPα2			
R99Q	0.2315	likely benign	Surface
E232D	0.1309	likely benign	Surface
E232K	0.2572	likely benign	Surface
R246Q	0.1286	likely benign	Stability
R385Q	0.0808	likely benign	Stability
P439H	0.9001	likely pathogenic	Stability
D454N	0.0736	likely benign	Loop
D454Y	0.0972	likely benign	Loop
R551H	0.0802	likely benign	Surface
K560T	0.1399	likely benign	Surface
L683I	0.0694	likely benign	Loop
G692V	0.3277	likely benign	Loop
G692W	0.6096	likely pathogenic	Loop
E707K	0.4844	ambigous	Loop
P771L	0.0662	likely benign	Loop
S790L	0.0502	likely benign	Loop
R888C	0.0794	likely benign	Loop
R888S	0.1767	likely benign	Loop
P1072L	0.3627	ambigous	β Hug Binding
P1072S	0.3614	ambigous	β Hug Binding
P1119S	0.1139	likely benign	β Hug Binding
P1372H	0.658	likely pathogenic	Stability
P1372L	0.6238	likely pathogenic	Stability
E1404K	0.8282	likely pathogenic	Stability
S1417F	0.4812	ambigous	Stability
S1497L	0.0954	likely benign	Hug domain
R1501C	0.0667	likely benign	Hug domain
R1501P	0.0837	likely benign	Hug domain
G1590E	0.068	likely benign	Hug domain
G1590K	0.103	likely benign	Hug domain
R1738H	0.8389	likely pathogenic	Ral Binding
A1857T	0.2198	likely benign	Heterodimerization
RalGAPβ			
C86R	0.998	likely pathogenic	Stability
G134D	0.192	likely benign	Surface
G134S	0.075	likely benign	Surface
K408E	0.346	ambigous	Loop
L446F	0.7678	likely pathogenic	Loop
P568S	0.0751	likely benign	Stability
S613F	0.6525	likely pathogenic	Stability
S620F	0.738	likely pathogenic	Stability
R717C	0.3867	ambigous	Loop
R717H	0.2507	likely benign	Loop
G721D	0.7047	likely pathogenic	Loop

G721S	0.0924	likely benign	Loop
S917G	0.236	likely benign	α Hug binding
R1010C	0.22	likely benign	Hug Domain
R1010H	0.1713	likely benign	Hug Domain
V1032A	0.9069	likely pathogenic	Hug Domain
V1032M	0.9322	likely pathogenic	Hug Domain
E1060K	0.1533	likely benign	Hug Domain
L1154F	0.1729	likely benign	Stability
P1159L	0.358	ambigous	Stability
S1220T	0.0836	likely benign	Stability
S1220Y	0.2139	likely benign	Stability

Supplementary references

1. Yang, H. *et al.* Structural insights into TSC complex assembly and GAP activity on Rheb. *Nat Commun* 12, 339 (2021).
2. Hansmann, P. *et al.* Structure of the TSC2 GAP Domain: Mechanistic Insight into Catalysis and Pathogenic Mutations. *Structure* 28, 933–942 (2020).
3. Scrima, A., Thomas, C., Deaconescu, D. & Wittinghofer, A. The Rap-RapGAP complex: GTP hydrolysis without catalytic glutamine and arginine residues. *EMBO J* 27, 1145–1153 (2008).
4. Gao, J. *et al.* Integrative analysis of complex cancer genomics and clinical profiles using the cBioPortal. *Sci Signal* 6, pl1 (2013).
5. Cerami, E. *et al.* The cBio cancer genomics portal: an open platform for exploring multidimensional cancer genomics data. *Cancer Discov* 2, 401–4 (2012).
